# CCR2 and TLR3: noninvasive macrophage-associated biomarkers of acute renal transplant rejection

**DOI:** 10.1080/0886022X.2025.2532856

**Published:** 2025-07-27

**Authors:** Shili Wang, Xingyu Pan, Jinpu Peng, Moudong Wu, Xiong Zhan, Wei Wang, Guohua Zhu, Nini An, Jun Pei

**Affiliations:** a Department of Pediatric Urology, Guizhou Branch of Shanghai Children’s Medical Center Shanghai Jiaotong University School of Medicine, Guiyang, China; b Department of Pediatric surgrey, Guizhou Provincial People’s Hospital, Guiyang, China

**Keywords:** Renal transplantation, noninvasive, acute rejection, macrophages, immune infiltration

## Abstract

**Background:**

The study aims to investigate the role and significance of macrophage-related genes in peripheral blood during acute renal transplant rejection.

**Methods:**

Based on the dataset GSE15296, differential genes (DEGs) were intersected with macrophage-associated genes (MAGs), which defined as macrophage-associated genes (DEMAGs). Subsequently, GO and KEGG enrichment analyses were performed on DEMAGs. The PPI protein interaction network and machine learning were applied to identify Hub genes, which were also validated by GSE46474, an external validation set. We constructed a rat model of acute rejection of kidney transplantation and sequenced the transcriptome of the serum, and using the sequencing results, we analyzed the expression levels of the Hub gene. Then, a correlation analysis was carried out based on the Hub genes and different immune cell infiltration levels. Finally, GRNdb, miRDB, and GeneCards databases were applied to study the transcription factors, miRNAs, and regulatory drugs of Hub genes, respectively.

**Results:**

The results of the Cibersort analysis revealed that M2-type macrophages were expressed higher in the Normal group. In addition, the results of the correlation analysis suggested that the expressions of Hub genes were negatively correlated with M1-type macrophages and positively correlated with M2-type macrophages.

**Conclusion:**

A diagnostic model of the macrophage-associated acute renal transplant rejection in peripheral blood was constructed based on CCR2 and TLR3, which can accurately diagnose the biological alterations of acute kidney transplant rejection. Meanwhile, these two Hub genes may become potential therapeutic targets in acute rejection of kidney transplantation.

## 1. Introduction

For patients with end-stage renal disease, renal transplantation can not only improve patients’ health and quality of life but also prolong their life expectancy. However, acute injury of the graft, acute/chronic rejection, and infection during the renal transplantation often result in impairment of the transplanted renal function, leading to the need for secondary renal transplantation in some patients, which in severe cases will lead to the death of the patient [1]. Among the influencing factors, acute renal transplant rejection is considered to be one of the important factors affecting the function of transplanted kidneys [2]. To improve the efficacy of renal transplantation, it is necessary to adopt accurate graft assessment techniques and innovative therapies to gain a deeper understanding of the mechanism of acute renal transplant rejection, thus better achieving personalized treatment for renal transplant patients.

Macrophages are ubiquitous cell types that are widely found in tissues and organs. Macrophages were believed to be the first line of defense against infection because their short-lived physiological function is phagocytosis [3]. In recent years, it has been discovered that macrophages, which are considered to be an important component of the innate immune response that collaborates with other innate immune cells, appear to play a crucial role in the immune response, increasing their ability to respond to stimuli [4]. With the increasing research on macrophage biology, it has been found that different types of macrophages exist in the kidney, among which the differentiation of M1-type and M2-type macrophages has attracted extensive attention. Currently, it has been found that M1-type macrophages can produce a variety of inflammatory factors, such as IFN-γ, IL-1β, and TNF-ɑ, as well as inducible nitric oxide synthase (iNOS) to promote the production of reactive oxygen species (ROS), resulting in renal tissue damage [5,6]. M2-type macrophages, on the other hand, can produce a variety of anti-inflammatory factors, such as IL-10 to promote increased expression of arginase-1 (Arg-1) and deplete L-arginine, thereby reducing the occurrence of T cell proliferative effects [7]. Therefore, inducing differentiation of different types of macrophages seems to have a good application in the protection of transplanted renal function.

At present, the diagnosis of acute renal transplant rejection often requires renal biopsy [8], which may cause further damage to the function of the transplanted renal, as well as increase the risk of bleeding and infection. Therefore, the identification and prediction of noninvasive markers of acute renal transplant rejection as well as the construction of a diagnostic model are now receiving widespread attention. In the study, we provided new ideas for the future diagnosis and prediction of noninvasive acute renal transplant rejection through the identification of macrophage-related markers in the peripheral blood of patients with acute renal transplant rejection and the construction of a diagnostic model.

## 2. Materials and methods

### 2.1. Data set sources and processing

First, two peripheral blood sequencing datasets related to acute renal transplant rejection from the GEO database (Gene Expression Omnibus, http://www.ncbi.nlm.nih.gov/geo) were selected for the study, with the Dataset GSE15296 (GPL570, Affymetrix Human Genome U133 Plus 2.0 Array) containing 51 patients with acute rejection (AR) and 24 patients without acute rejection (Normal), and the Dataset GSE46474 (GPL570, Affymetrix Human Genome U133 Plus 2.0 Array) containing 20 patients with acute rejection (AR) and 20 patients without acute rejection (Normal). The dataset GSE15296 was used as the training set and the dataset GSE46474 as the external validation set. All datasets were preprocessed for normalization by the ‘Sangerbox online analysis tool’ (www.sangerbox.com) [9].

Secondly, we searched the Molecular Signature Database (MSigDB, http://www.gseamsigdb.org/gsea/msigdb/index.jsp) to obtain a total of 240 macrophage-associated genes (MAG), with ‘macrophage’ as the keyword and the C5- Gene Ontology (GO) as the filtering condition.

### 2.2. Enrichment analysis of macrophage-associated genes

The Gene Ontology (GO) and Kyoto Encyclopedia of Gene and Genes (KEGG) enrichment analysis of macrophage-associated genes (MAG) were performed using the Hiplot (https://hiplot.org) online analysis tool, with the GO enrichment analysis including biological processes (BP), cellular components (CC), and molecular functions (MF), and adj. *p* < 0.05 indicates the threshold for screening the major enrichment functions and pathways of MAG. P values were corrected using the Benjamini-Hochberg (BH) method. Hiplot is a visualization application loaded with 240+ biomedical data, covering a wide range of functions, such as basic statistics, multi-omics, regression, clustering, reduced-dimension, meta-analysis, survival analysis, and risk modeling [10].

### 2.3. Identification and functional analysis of differentially expressed macrophage-related genes

In the training set GSE15296, the differentially expressed genes (DEGs) between the acute rejection (AR) and no acute rejection (Normal) groups were identified. In order to be able to obtain more DEGs, we set the criteria for the identification of DEGs to Log2Fold absolute value > 0.5 and adj. *p* < 0.05. P values were corrected using the FDR method. To further identify differentially expressed macrophage-associated genes in the peripheral blood in acute renal transplant rejection, the DEGs were intersected with MAG, with the intersection genes as differentially expressed macrophage-associated genes (DEMAGs) in peripheral blood. To further clarify the potential biological functions and molecular mechanisms of DEMAG, we again analyzed DEMAG by GO and KEGG enrichment using the Hiplot online analysis tool, with adj. *p* < 0.05 indicating statistically significant. P values were corrected using the Benjamini-Hochberg (BH) method.

### 2.4. Identification and validation of Hub genes

To further explore the key genes associated with peripheral blood macrophages in acute renal transplant rejection, the PPI protein interaction network and machine learning were combined to identify Hub genes. First, a PPI network of DEMAG protein interactions was constructed using the online bioresource database String (http://www.string-db.org/) for visualization by the Cytoscape software. Subsequently, the ‘MCC, MNC, EPC, Degree and Closeness’ algorithms in the cytoHubba plug-in of the Cytoscape software were applied to rank DEMAG, with the top 10 genes used as the key genes for each algorithm. To further improve the accuracy of the key genes, the top 10 genes of the five different algorithms were intersected, with the intersection genes as ‘PPI-key genes’. Then, the ‘LASSO regression analysis’ in the ‘Sangerbox online analysis tool’ was utilized to identify key genes in the DEMAG, which were defined as ‘LASSO-key genes’. Finally, we ranked the importance of DEMAG by the ‘Random Forest’ algorithm in the ‘Ouyi Cloud Platform’ (www.cloud.oebiotech.cn), with the top 10 genes defined as ‘Random Forest - Key Genes’. Further, the screened key genes by the above three different algorithms were intersected, with the intersection genes defined as ‘Hub Genes’. A diagnostic model for the prediction of acute renal transplant rejection was constructed by using the ‘nomogram model’ in the Hiplot online analysis tool to predict acute renal transplant rejection by the Hub genes. The diagnostic model construction process was performed based on multifactor logistic regression analysis. The receiver operating characteristic curves (ROC) and calibration curve used to evaluate the diagnostic performance of the Hub genes and the predictive ability of the nomogram model. Finally, the expressions of Hub genes in the Normal and AR groups in the training set were statistically analyzed, with *p* < 0.05 indicating considered statistically significant.

To further clarify the diagnostic value of the Hub genes, a ‘nomogram model’ of the Hub genes was constructed in the external validation set GSE46474, and the diagnostic performance of the Hub genes and the prediction ability of the nomogram model were evaluated using the ROC curve and the calibration curve, with the AUC > 0.7 in the ROC curve considered to be of diagnostic value [11]. The diagnostic model construction process was still performed based on multifactor logistic regression analysis. Finally, the expressions of Hub genes were statistically analyzed, with *p* < 0.05 indicating statistically significant.

### 2.5. Construction of animal models

We used 12 adult male Sprague-Dawley (SD) rats and 4 Wistar rats, weighing between 250 and 300 g. Recipients were selected from SD rats, which were randomly divided into two groups of four rats each; Donors were categorized into SD rats and Wistar rats, four each; Donors from SD rats were transplanted into recipients from SD rats, which we defined as the kidney transplantation without acute rejection group (Syn group); Donors from Wistar rats were transplanted into recipients from SD rats, which we defined as the acute rejection group for kidney transplantation (Allo group). The specific process of kidney transplant modeling remains consistent with our previous study [12]. The SD rats were purchased from Chongqing Medical University Laboratory Animal Center (SYXK[YU] 2022-0016, Chongqing, China); the Wistar rats were purchased from Beijing Vital River Laboratory Animal Technology Co., Ltd. (SCXK (Beijing) 2021-0006).

### 2.6. Transcriptome sequencing

We performed transcriptome sequencing of serums from four rats in each group in this experiment, and performed statistical analysis of the mRNA expression of Hub gene in the sequencing results to verify the expression level of Hub gene in serums. An Illumina NovaSeq 6000 platform from LC-Bio was used in part for high-throughput sequencing (Hangzhou, China).

### 2.7. Immune cell infiltration and correlation analysis

The ‘immune infiltration’ in the Sangerbox online analysis tool was used to calculate the proportion of 22 immune cells in the training set of GSE15296 samples and compare the expression levels of 22 immune cells in the AR group and the Normal group, so as to explore the changes of the immune cells in peripheral blood during the acute renal transplant rejection. In order to understand the correlation between the Hub genes and the 22 human immune cells, the ‘Sample Correlation Analysis’ in the ‘Ouyi Cloud Platform’ (www.cloud.oebiotech.cn) was used to calculate the correlation between the 22 immune cells and the Hub genes, and a lollipop plot was used to visualize the correlation analysis, with *p* < 0.05 indicating statistically significant.

### 2.8. Construction and functional analysis of hub gene-transcription factor (TF) network

To explore the regulatory network between transcription factors (TFs) and Hub genes, we explored the TFs associated with Hub gene regulation using the GRNdb database, an online analysis site for studying the correlation between transcription factors and target genes based on large-scale RNA-seq data [13]. We intersected the transcription factors of each Hub gene, with the intersected regarded as the common TFs capable of regulating all Hub genes. In order to further investigate the biological functions and mechanisms of the common TFs of the Hub genes, we analyzed them with GO and KEGG enrichment, with adj. *p* < 0.05 indicating statistically significant. P values were corrected using the Benjamini-Hochberg (BH) method. Next, we further studied the differentially expressed TFs in peripheral blood in acute renal transplant rejection and intersected the differential expressed genes in the training set GSE15296 with the common TFs of the Hub genes again, with the intersection genes defined as the differentially expressed TFs in the peripheral blood in acute renal transplant rejection. In order to understand the correlation between the Hub genes and the differentially expressed TFs in the peripheral blood, the correlation between the differential TF and the Hub genes was calculated using the ‘sample correlation analysis’ in the ‘Ouyi cloud platform’ (www.cloud.oebiotech.cn) and the correlation results were visualized with a lollipop chart, with *p* < 0.05 indicating statistically significant.

### 2.9. Hub gene-miRNA regulatory network construction

The miRDB database (https://mirdb.org) is an online database for miRNA target prediction and functional annotation [14], which was used to predict miRNA targeting and binding to Hub genes. In addition, the cytoscape software was used to construct the miRNA-Hub gene network.

### 2.10. Identification of hub gene-related drugs

The GeneCards database (https://www.genecards.org/) is a comprehensive database that provides information on all annotated and predicted human genes [15], in which the ‘Drugs’ section provides drugs or compounds from multiple sources such as DrugBank, ClinicalTrials, ApexBio, DGIdb, IUPHAR, and Novoseek. Also, the GeneCards database was applied to explore potential drugs or compounds related to the Hub genes. In addition, the PubChem database (https://pubchem.ncbi.nlm.nih.gov/) was utilized to display 3D structure diagrams of related drugs or compounds [16].

### 2.11. Statistical analysis

Prism software (GraphPad Software, La Jolla, CA) was used for statistical analysis of experimental data, and unpaired t-tests were used for comparisons between groups, with **** indicating *p* < 0.0001, *** indicating *p* < 0.001, ** indicating *p* < 0.01, and * indicating *p* < 0.05.

## 3. Results

### 3.1. Macrophage-associated gene (MAG) enrichment analysis

**Figure 1.**
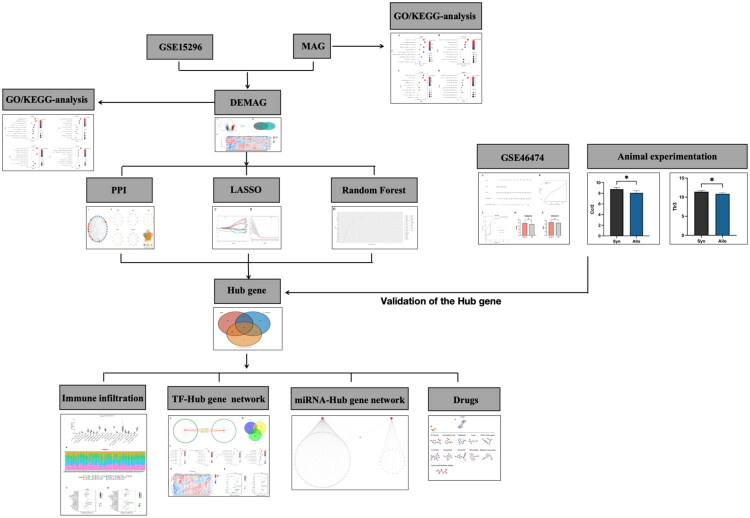
Research flowchart.

**Figure 2.**
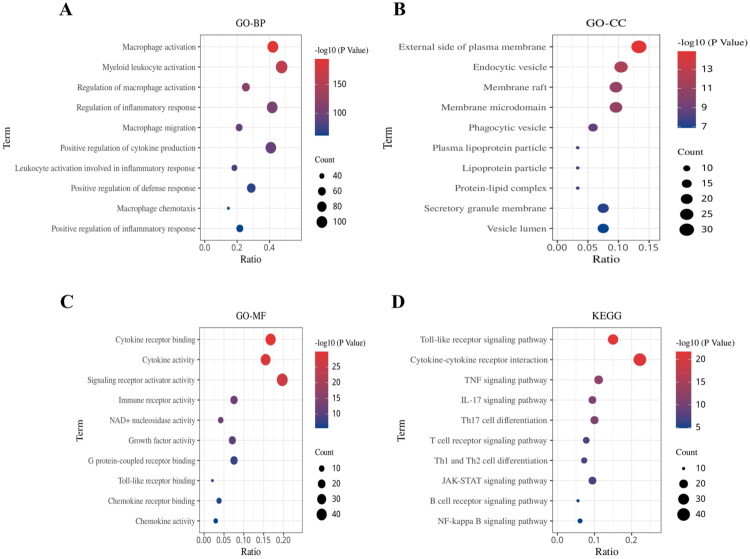
Macrophage-associated gene (MAG) enrichment analysis. A represents biological process (BP) in GO enrichment analysis; B represents cellular component (CC) in GO enrichment analysis; C represents molecular function (MF) in GO enrichment analysis; and D represents KEGG enrichment analysis.

The flow chart of the study is shown in Figure 1. A total of 240 macrophage-associated genes were found by searching the MSigDB database. The GO and KEGG enrichment analysis by Hiplot showed that MAG was mainly enriched in macrophage activation, bone marrow leukocyte activation, regulation of inflammatory response, macrophage infiltration, and regulation of cytokine production (Figure 2A) in biological process (BP); MAG was mainly enriched in cytoplasmic membranes, endocytosed membrane vesicles, membrane microstructural domains, and phagocytic vesicles in cellular component (CC) (Figure 2B); MAG were mainly enriched in cytokine receptor binding, cytokine activation, chemokine activation, immune receptor activation, and Toll-like receptor binding in molecular function (MF) (Figure 2C). KEGG enrichment analysis revealed that MAG was mainly enriched in the Toll-like receptor signaling pathway, cytokine-cytokine receptor interaction, TNF signaling pathway, IL-17 signaling pathway, and T cell receptor signaling pathway (Figure 2D). Therefore, it was found that the biological functions of MAG are closely related to the regulation and activation of macrophages and the immune response.

### 3.2. Identification and enrichment analysis of differentially expressed macrophage-associated genes (DEMAG)

**Figure 3.**
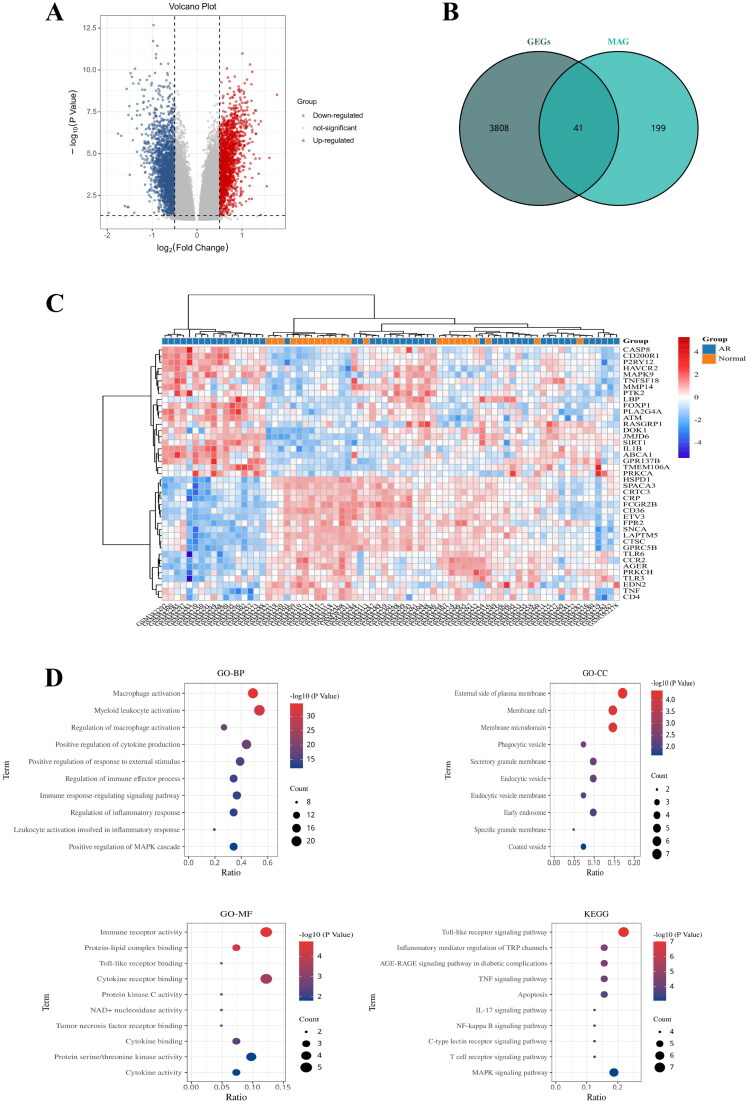
Identification and enrichment analysis of differentially expressed macrophage-associated genes (DEMAG). A represents the differentially expressed gene expression volcano plot in the training set GSE15296; B represents the veen plot of the intersection of differentially expressed genes (DEGs) and macrophage-associated genes (MAGs); C represents the heatmap of differentially expressed macrophage-associated genes (DEMAG); D represents the GO and KEGG enrichment analysis plot of DEMAG, where biological process (BP), cellular component (CC), and molecular function (MF) are included in the GO enrichment analysis.

A total of 3,849 differentially expressed genes (DEGs) were identified in the training set GSE15296, of which 2,216 were downregulated and 1,633 were upregulated in acute renal transplant rejection (Figure 3A). After intersecting the DEGs with the 240 MAGs obtained from the MSigDB database, a total of 41 genes were obtained to be defined as differentially expressed macrophage-related genes (DEMAGs) (Figure 3B,C). In addition, GO and KEGG enrichment analyses were performed to further clarify the potential biological functions and mechanisms of the DEMAGs, with the GO enrichment analysis results showing that DEMAG was mainly related to macrophage activation and regulation, chemokine regulation, inflammatory response, and immune response (Figure 3D), and the KEGG enrichment analysis results revealing that DEMAG was mainly enriched in Toll-like receptor signaling pathway, TNF signaling pathway, apoptotic response, NF-KB signaling pathway, and T cell receptor signaling pathway (Figure 3D). Therefore, DEMAG is mainly associated with macrophage regulation, inflammation, and immune system response.

### 3.3. Identification of Hub genes

**Figure 4.**
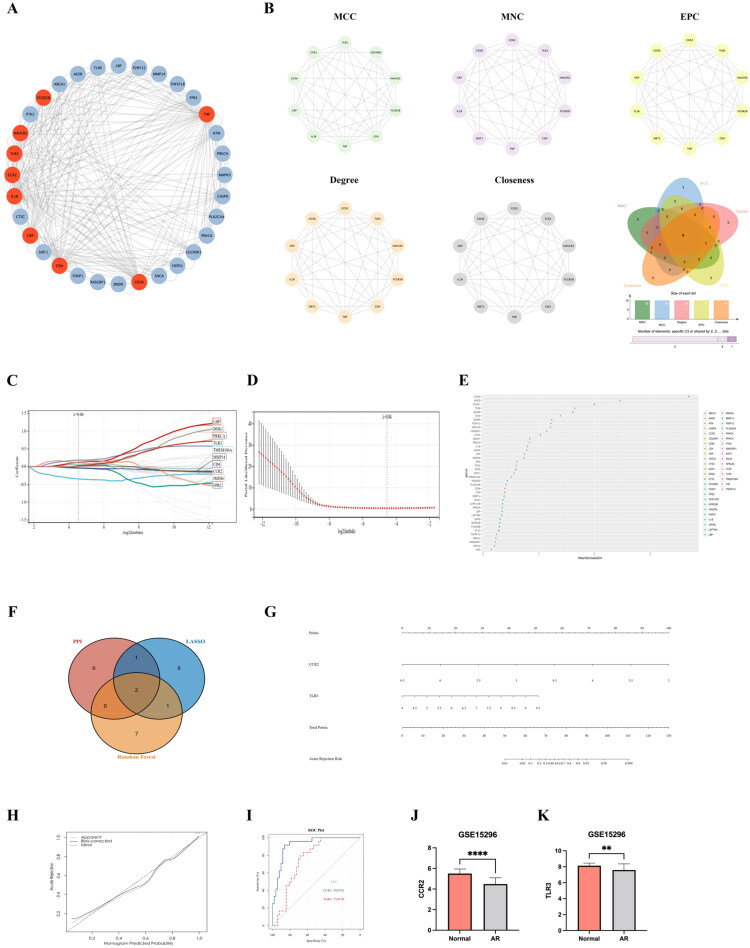
Identification of Hub genes and construction of diagnostic models. A represents the PPI protein interactions network visualized by the cytoscape software, where the genes after the intersection of 5 different algorithms are shown in red; B represents the top 10 genes and the veen plot of the intersection of 5 algorithms obtained by using ‘MCC, MNC, EPC, degree, and closeness’ algorithms with the cytoHubba plugin; C-D represents the LASSO regression analysis, which finally obtained the 10 key genes when the lambda value was 0.04; E represents the random Forest plot of DEMAG, where the top 10 genes are the key genes; F represents the veen plots of the intersection of the three different algorithms; G represents the ‘nomogram model’ constructed based on Hub genes; H represents the calibration curve of the nomogram model; I represents the ROC curve of Hub genes; J represents the expression statistics of Hub gene CCR2 in the training set GSE15296; and K represents the expression statistics of Hub gene TLR3 in the training set GSE15296.

In the study, the PPI protein interaction network and machine learning (LASSO regression analysis and random forest) were applied to identify Hub genes. First, 41 DEMAGs were entered into the String database to construct a PPI protein interaction network, which was then visualized using the Cytoscape software (Figure 4A). Next, the ‘MCC, MNC, EPC, Degree and Closeness’ algorithms of the cytoHubba plug-in were utilized to calculate the top 10 genes in the different algorithms (Figure 4B), with a total of 9 intersection genes obtained after the intersection between the genes obtained from the five different algorithms defined as ‘PPI-key genes’, namely, CD36, CD4, CRP, IL1B, CCR2, TLR3, HAVCR2, FCGR2B, and TNF (Figure 4A). Then, the ‘LASSO regression analysis’ in the Sangerbox platform was used to obtain 10 key genes when the Lambda value was set to 0.04, which were defined as ‘LASSO-key genes’, namely, LBP, DOK1, PRKCA, TLR3, TMEM106A, MMP14, CD4, CCR2, JMJD6, and FPR2 (Figure 4C,D). Finally, the ‘Random Forest’ algorithm of the Ouyi Cloud Platform was used to rank the DEMAGs, with the top 10 genes defined as ‘Random Forest-Key Genes’, namely, CCR2, AGER, FOXP1, TLR6, JMJD6, TLR3, CASP8, P2RY12, SPACA3, and CD200R1 (Figure 4E). To further improve the accuracy of the key genes, we intersected the key genes screened by the above three different algorithms to obtain CCR2 and TLR3, which were defined as Hub genes (Figure 4F).

A ‘nomogram model’ was constructed based on the two Hub genes (Figure 4G), with the calibration curve showing that the constructed nomogram diagnostic model had a better diagnostic efficacy (Figure 4H), and the AUC of the area under the ROC curve was greater than 0.7 (CCR2: AUC = 0.92; TLR3: AUC = 0.76) (Figure 4I). In addition, the statistical analysis of the Hub gene expressions in the AR group and the Normal group showed that the expression levels of CCR2 and TLR3 in the AR group were significantly lower than those in the Normal group (*p* < 0.05) (Figure 4J,K). Therefore, we believe that changes in peripheral blood of the Hub genes, comprising CCR2 and TLR3, may have potential predictive properties and diagnostic value for the development of acute rejection in kidney transplantation.

### 3.4. Validation of Hub genes

**Figure 5.**
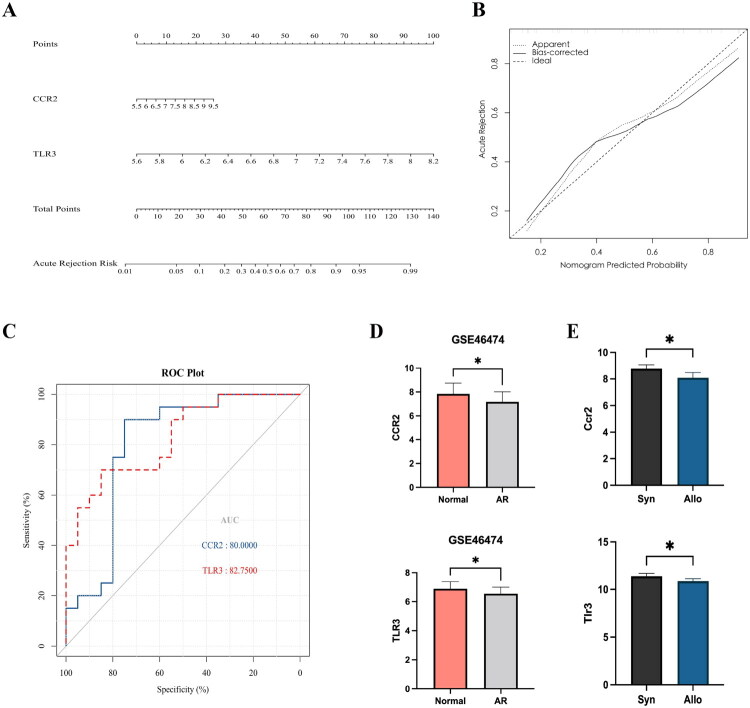
Validation of Hub genes. A represents the ‘nomogram model’ constructed by the Hub genes; B represents the calibration curve of the nomogram model; C represents the ROC curve of the Hub genes; D represents the expression statistics of the Hub gene CCR2 and TLR3 in the validation set GSE46474; E represents CCR2 and TLR3 gene expression results from serum transcriptome sequencing results in a rat model of acute rejection of kidney transplantation.

To verify the accuracy of the Hub genes, a ‘nomogram model’ was again constructed using the Hub genes CCR2 and TLR3 in the external validation set GSE46474 (Figure 5A), with the calibration curves showing that the ‘nomogram model’ constructed by CCR2 and TLR3 in the validation set had better diagnostic efficacy (Figure 5B), the AUC under the ROC curve was greater than 0.7 (CCR2: AUC = 0.8; TLR3: AUC = 0.83) (Figure 5C), and the expression trends of Hub genes CCR2 and TLR3 in the validation set were consistent with those in the training set, and the differences were statistically significant (Figure 5D). At the same time, we constructed a model of acute rejection of kidney transplantation in rats and sequenced the transcriptome of rat serum. Based on the sequencing results, we found that the expression levels of CCR2 and TLR3 were significantly lower in the Allo group compared with the Syn group, and the difference was statistically significant (*p* < 0.05), and the trend remained consistent with the results of the dataset analysis (Figure 5E). This suggests that changes in the Hub genes CCR2 and TLR3 in the peripheral blood of kidney transplantation may be potentially relevant to the development of acute rejection in kidney transplantation.

### 3.5. Immune cell infiltration and correlation analysis

**Figure 6.**
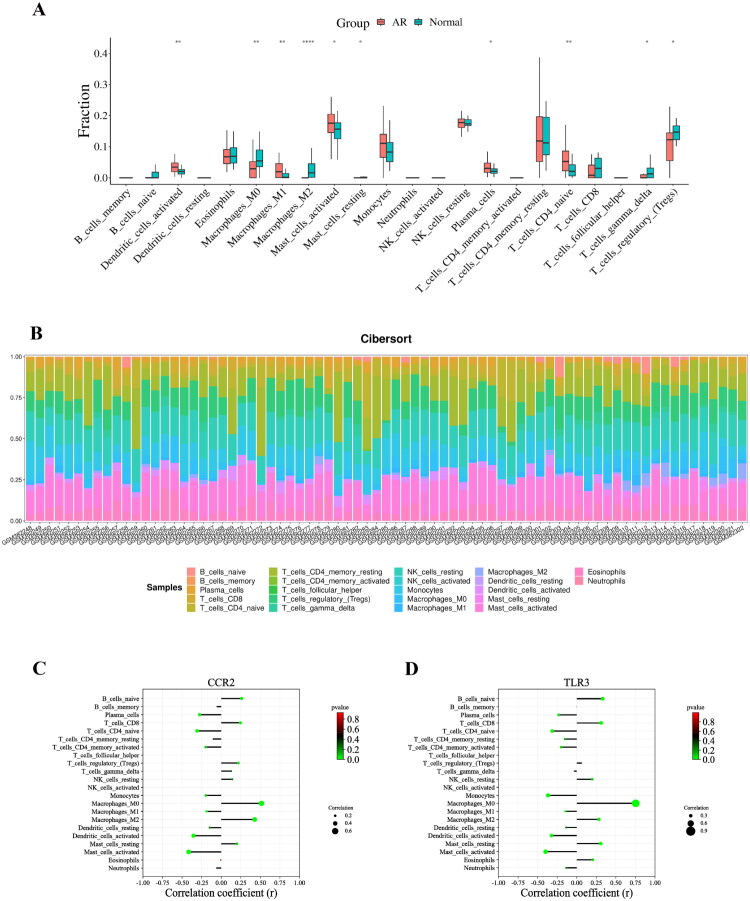
Immune cell infiltration and hub gene and immune cell correlation analysis. A represents a graph of the difference in expressions of 22 immune cells between the AR and normal groups assessed using cibersort; B represents a stacking diagram of expression levels of the 22 immune cells across samples; C represents a graph of correlation analysis of the hub gene CCR2 with the 22 immune cells; and D represents a graph of correlation analysis of the hub gene TLR3 with the 22 immune cells.

The exploration of expression levels of 22 types of immune cells in the human body by Cibersort showed that 10 immune cells had statistically different expressions between the AR and Normal groups (Figure 6A,B), implying that changes in immune cells may play a key role during acute renal transplant rejection. Among them, it was found that the expression of macrophage M1 was significantly higher in the AR group, but macrophage M2 was most significantly expressed in the Normal group (Figure 6A,B). Macrophage activation is thought to have an important role in graft function and the onset of rejection. Next, the correlation analysis between the Hub genes and 22 immune cells showed that the Hub genes CCR2 and TLR3 were correlated with a variety of immune cells, among which expressions of CCR2 and TLR3 were found to be negatively correlated with macrophage M1, but positively correlated with macrophage M2 (Figure 6C,D). This means that increased expressions of CCR2 and TLR3 will decrease the expression of macrophage M1 and increase the expression of macrophage M2.

### 3.6. Construction of hub gene-transcription factor (TF) network and functional analysis

**Figure 7.**
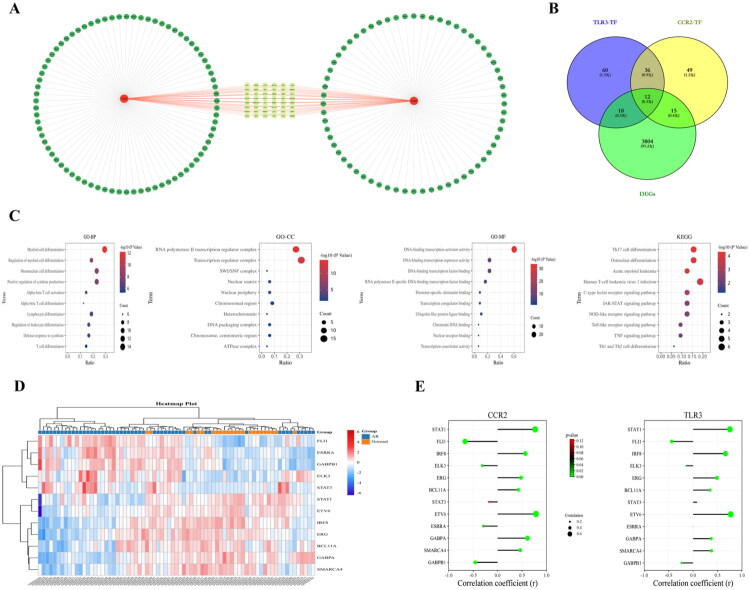
Construction of hub gene-transcription factor (TF) network and functional analysis. A represents the regulatory network diagram of hub genes and transcription factors (TFs); B represents the wayne diagram of the intersection of TFs of hub genes and DEGs; C represents the GO and KEGG enrichment analysis plot of the intersecting TFs, where biological processes (BP), cellular components (CC), and molecular functions (MF) were included in the GO enrichment analysis D represents the heat map of differential TF expression; and E represents the correlation expression between hub genes and differential TFs.

In order to further explore the regulatory mechanism of Hub genes in peripheral blood in acute renal transplant rejection, we predicted the transcription factors (TFs) of Hub genes with the help of the GRNdb database, with a total of 112 TFs found to regulate CCR2 and a total of 126 TFs found to regulate TLR3 (Figure 7A). The intersection of these TFs showed a total of 48 TFs can regulate both CCR2 and TLR3 (Figure 7B), based on which a Hub gene-TF regulatory network was constructed using the Cytoscape software (Figure 7A). To further understand the potential biological functions and regulatory mechanisms of the transcription factors, the GO enrichment analysis of the 48 TFs was performed to find that the intersecting TFs were mainly enriched for immune-regulation-related biological functions, such as regulation and infiltration of myeloid cells, infiltration of monocytes, production of regulating cytokine, and activation and infiltration of T cells. The KEGG enrichment analysis similarly revealed that immunomodulation-related pathways, such as the infiltration of Th17 cells, the Toll-like receptor signaling pathway, the infiltration of Th1 and Th2 cells, and the NOD-like receptor signaling pathway were activated. As a result, Hub gene-related TFs are mainly closely related to the immune regulation of the body (Figure 7C). Next, in order to understand the TFs with differential expression in the peripheral blood of acute renal transplant rejection, the intersected 48 TFs were intersected with differentially expressed genes to identify a total of 12 TFs, which were defined as differential TFs in the peripheral blood of acute renal transplant rejection (Figure 7B,D). These differential TFs were considered to be the key to regulating Hub genes in peripheral blood in acute renal transplant rejection. Therefore, the further correlation analysis between Hub genes and differential TFs showed that the expressions of TLR3 and CCR2 were associated with a variety of differential TFs (Figure 7E), providing a theoretical basis for the subsequent regulation of Hub genes.

### 3.7. Construction of Hub gene-miRNA network

**Figure 8.**
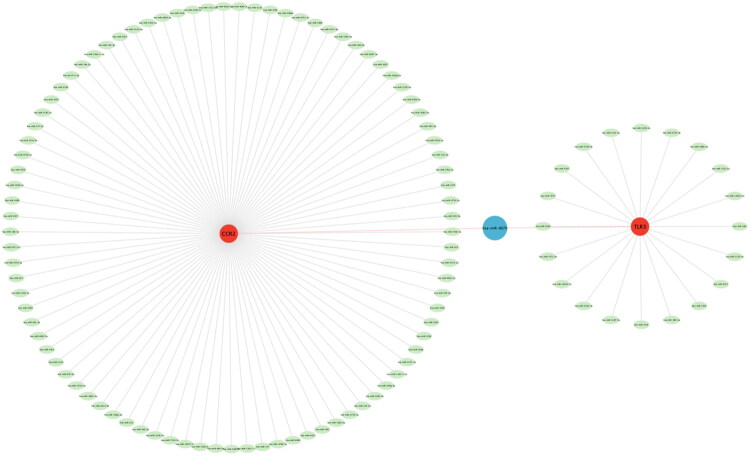
Construction of Hub gene-miRNA network. Red represents Hub genes, green represents miRNAs, and blue represents miRNAs that co-regulate two Hub genes.

The study on miRNAs that may regulate the Hub genes by the miRDB database revealed a total of 90 miRNAs associated with the CCR2 regulation (Figure 8) and a total of 21 miRNAs associated with the TLR3 regulation (Figure 8), and the hsa-miR-8070 could regulate both TLR3 and CCR2 (Figure 8). The Hub gene-miRNA regulatory network was again constructed using the Cytoscape software (Figure 8). This finding of ours provides a theoretical basis for the regulation of Hub genes during acute rejection of kidney transplantation, but subsequent more in-depth validation of its regulatory role in animal experiments is still needed.

### 3.8. Identification of potential drugs for the Hub genes

**Figure 9.**
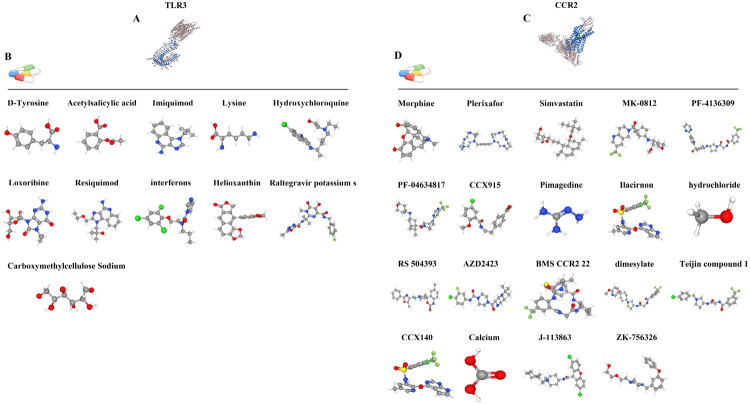
Hub gene-related regulatory drugs. A represents the gene structure of TLR3; B represents the TLR3-related regulatory drugs and 3D structure; C represents the gene structure of CCR2; D represents the CCR2-related regulatory drugs and 3D structure.

To further investigate relevant drugs that can regulate the Hub genes, the GeneCards database was applied to explore drugs or molecular compounds that may interact with the Hub genes. The results showed a total of 30 drugs or compounds regulated the Hub gene, of which 11 were associated with TLR3 regulation and 19 with CCR2 regulation (Figure 9A–D), with the 3D structures of the genes and drugs shown in Figure 9A–D. This provides a theoretical basis for the study of pharmacological regulation of Hub genes, but the effect in kidney transplantation AR still needs to be further investigated by subsequent experiments.

## 4. Discussion

Renal transplantation is currently the treatment of choice for end-stage chronic kidney disease (CKD), which can improve patients’ quality of life and mortality compared with dialysis [17]. However, it has been difficult to predetermine the success or failure of allogeneic transplantation in renal transplant recipients to date. Studies have shown that assessment of glomerular filtration rate or proteinuria may not predict future allograft failure [18]. Some scholars have established a new renal graft failure scoring criterion in order to improve the accuracy of predicting allogeneic transplantation failure, including the recipient’s age, gender, creatinine, donor creatinine, and recipient’s serum creatinine at 3 and 12 months after transplantation [18], which, however, largely depends on long-term follow-up of renal function and requires invasive biopsies of allograft renal, largely increasing the risk of transplant renal injury [18]. In kidney transplantation, the ideal biomarker should be able to quickly, accurately, inexpensively, and noninvasively recognize early allograft injury and discriminate the type of injury. As a result, noninvasive biomarkers are now receiving widespread attention. For example, donor-specific anti-HLA antibodies (DSA), donor-derived cell-free DNA (dd-cfDNA), and the IFNγ-induced chemokines CXCL9 and CXCL10 are currently well studied [19]. However, the above biomarkers still have some limitations in the monitoring of AR in kidney transplantation and the thresholds determined and the sensitivity and specificity of the diagnosis varied in different studies. Therefore, to date, no diagnostic criteria can be identified. Renal biopsy remains the current ‘gold standard’ [20]. In order to help achieve early detection of acute rejection in kidney transplantation, while also avoiding the damage caused by invasive renal biopsy, we still need to further explore novel noninvasive biomarkers to provide new options for the detection of acute rejection in kidney transplantation.

In the study, the differentially expressed genes (DEGs) in the peripheral blood during acute renal transplant rejection were first identified by intersecting the DEGs with macrophage-associated genes (MAGs), with the intersection genes defined as differentially expressed macrophage-associated genes (DEMAGs) and functionally analyzed to explore the potential biological functions. Based on the expression profile of DEMAG, a diagnostic model of peripheral blood for acute renal transplant rejection consisting of two Hub genes was constructed, which has accurate diagnostic performance and potential to predict acute renal transplant rejection. In addition, the enrichment analysis targeting transcription factors (TFs) upstream of the two Hub genes and the exploration of the miRNAs regulating the Hub genes were performed for the understanding of the transcriptional regulation mechanism of the Hub genes. Finally, the regulatory drugs of Hub genes were predicted to provide a theoretical basis for the selection of therapeutic drugs for acute renal transplant rejection.

First, a total of 240 macrophage-associated genes (MAGs) were obtained from the MSigDB database and previous studies were performed with GO and KEGG enrichment analyses to further explore the potential biological functions and mechanisms of MAGs, with the results showing that MAGs were mainly enriched in macrophage activation, myeloid leukocyte activation, regulation of inflammatory responses, macrophage infiltration, and production of regulating cytokine. It was also found that pathways related to immune response, such as the Toll-like receptor signaling pathway, TNF signaling pathway, IL-17 signaling pathway, and T-cell receptor signaling pathway were activated, indicating a close correlation between macrophage activation and immune response. Some scholars have found that cancer immunotherapy can be improved by inhibiting immune checkpoints in macrophages [21], and the blockage or activation of immune checkpoints centered on macrophages provides more possibilities for tumor immunotherapy [21]. In addition, glucomannan can take effect by activating the immune response of macrophages, increasing the secretion of immune effector molecules, enhancing phagocytosis of macrophages, and promoting the expression of M1-type macrophages [22]. Therefore, it can be seen that the immune function associated with macrophages is considered to be one of the best targets for intervention in a variety of diseases because of its correlation with the outcome of a variety of diseases.

Further, differentially expressed genes (DEGs) and MAGs in peripheral blood in acute renal transplant rejection were intersected to explore the changes in macrophage-associated genes in peripheral blood during acute renal transplant rejection, with a total of obtained 41 intersection genes defined as differentially expressed macrophage-associated genes (DEMAGs) in peripheral blood in acute renal transplant rejection. Next, the exploration of the biological functions and mechanisms of DEMAG using GO and KEGG enrichment analyses showed that DEMAG was mainly closely related to the functions of macrophage regulation, inflammation, and immune system response. This implies that macrophage activation during acute renal transplant rejection may have an impact on the outcome of renal transplantation by regulating inflammation as well as immune responses. It has been found that SLAMF8 induces acute renal transplant rejection through the TLR4 signaling pathway, leading to the expression of M1-type macrophages, which promotes the enhancement of inflammatory response [23]. It has been also found that IGT can alleviate acute renal transplant rejection by inhibiting KLF4-regulated M1-type macrophage polarization and attenuating the endothelial cell injury mediated by DSA [24]. Therefore, we believe that macrophage regulation plays an important role in the development of acute renal transplant rejection, and the identification of macrophage-related markers has significant potential for the prediction and treatment of rejection.

With the advantage of synthesizing the dual screening results of biological and mathematical methods to produce key genes with important biological and diagnostic roles, the method of combining the PPI protein interaction network and machine learning was carried out to screen Hub genes in our study. Ultimately, CCR2 and TLR3 were identified as macrophage-associated Hub genes in the peripheral blood of acute renal transplant rejection. The ROC curves and diagnostic model demonstrated that the Hub gene may have potential predictive value for acute rejection of kidney transplantation. The utilization of the external validation set also reaffirmed the possible significance of CCR2 and TLR3 in the diagnosis of acute rejection in kidney transplantation. It has been noted that the expression of CCR2 in renal transplant recipients is associated with delayed recovery of graft function before receiving renal transplantation [25], which implies that increased expression of CCR2 might exacerbate graft injury and lead to impaired graft function [25]. But to our surprise, the expression level of CCR2 in the peripheral blood of patients with acute rejection in the study was significantly lower than that of patients without acute rejection, which seems to be contrary to previous findings. A detailed understanding of the origin and developmental process of CCR2 indicated that it can be divided into two main groups: CCR2- and CCR2 + [26], with CCR2- considered to be an important participant and mediator in the process of tissue repair [26]. But unfortunately, this subgroup tends to be depleted during inflammation and repair, leading to an increased production of pro-inflammatory CCR2+ macrophages, which promotes inflammatory responses and exacerbates tissue damage [27]. In addition, CCR2-macrophages were again found to promote tissue repair and remodeling in rejection after cardiac transplantation, because a decrease in CCR2-macrophages increased the incidence of rejection after cardiac transplantation [26,27]. Therefore, we hypothesized that the reduced expression of CCR2 in patients with acute renal transplant rejection is mainly attributed to the depletion of the CCR2 subgroup, which leads to the development of acute rejection. Toll-like receptor 3 (TLR3) is thought to play a key role in both innate and adaptive immune responses [28]. TLR3, which is also often considered a prognostic and diagnostic marker for a wide range of tumors, is closely related to the immune microenvironment of tumors [29]. TLR3 is one of the TLR family members, which plays an important role in macrophage activation and polarization. In the LPS-induced *in vitro* model study, the TLR family can play an anti-inflammatory role by triggering mitophagy and inhibiting the inflammatory response through mtROS and mtRES signaling molecules [30]. However, there is currently no relevant study on the mechanism of TLR3 in renal transplant rejection at present, and we found in the study that the expression of TLR3 was reduced in the acute renal transplant rejection group, which might be related to the different directions of macrophage polarization.

To further explore the infiltration of immune cells during acute renal transplant rejection, the Cibersort was used to analyze the expression levels of 22 immune cells, with the results showing that the expression levels of 10 immune-related cells differed significantly during acute renal transplant rejection, among which M1-type macrophage expression significantly increased in the AR group and M2-type macrophage expression significantly increased in the Normal group. Macrophages are important immune cells involved in a range of inflammatory and autoimmune diseases through *in vivo* specific (cellular immunity) or nonspecific defense (innate immunity) [31]. With the highly plastic, heterogeneous, and pluripotent, macrophages can differentiate into different phenotypes and perform specialized functions in different microenvironments [32]. Based on their activation status and function, macrophages can be mainly classified into classically activated macrophages (M1) and alternatively activated macrophages (M2) [33], with M1-type macrophages participating in the immune response through the secretion of pro-inflammatory cytokines and chemokines and play a role in immune surveillance [23,34], while M2-type macrophages secreting anti-inflammatory cytokines, such as IL-4, IL-10 and TGF-β, as well as down-regulating the immune response to control immunomodulation and tissue remodeling [35], indicating increased expression of M1-type macrophages may be critical in causing acute renal transplant rejection. Previous studies have already found that activation of M1-type macrophages leads to the production of pro-inflammatory cytokines (TNF-α, IFN-ƴ, and IL-6), contributing to graft dysfunction and rejection [23,34]. In contrast, activation of M2-type macrophages will produce anti-inflammatory factors that are involved in the host defense response, tissue repair, and immune regulation, thereby improving the long-term survival of grafts [35]. In order to clarify the correlation between Hub genes and immune cells, the correlation analysis was performed to show that CCR2 and TLR3 were correlated with the expressions of various immune cells, which were negatively correlated with the expression of M1-type macrophages and positively correlated with the expression of M2-type macrophages. This implies that the secretion of M1-type macrophages will be increased when the expression of CCR2 and TLR3 is decreased. In combination with the previous finding of decreased expressions of CCR2 and TLR3 in the AR group, we hypothesized that the down-regulation of CCR2 and TLR3 expressions in the peripheral blood of renal transplantation patients will induce the polarization of M1-type macrophages and the secretion of anti-inflammatory factors, resulting in the occurrence of acute rejection of tissues.

The transcriptional regulatory mechanisms of organisms are diverse and complex, so regulating the activity of certain transcription factors (TFs) might be able to change the expression levels of genes and play a key role in the treatment of diseases [36]. In order to further understand the regulatory mechanism of Hub genes, the prediction of the TFs of Hub genes by GRNdb database was carried out to find that there were 112 TFs capable of regulating CCR2 and 126 transcription factors regulating TLR3, among which there were a total of 48 TFs capable of regulating the expressions of both CCR2 and TLR3. Next, the enrichment analysis of the 48 TFs showed that their functions were mainly related to the regulation of myeloid cells, monocyte infiltration, cytokine regulation, and activation of immune cells. It was also found that a large number of immune-regulation-related pathways were activated, which again demonstrated that the regulation of Hub genes is closely related to macrophage infiltration and immunomodulation. Further, the DEGs of acute renal transplant rejection were intersected with 48 TFs to clarify the TFs associated with Hub gene regulation in the peripheral blood of acute renal transplant rejection, with a total of obtained 12 intersected TFs defined as differential TF, which might play a critical role in regulating the Hub genes in the peripheral blood of patients with acute renal transplant rejection. Finally, the correlation analysis between Hub genes and differential TFs suggested that Hub genes were correlated with most of the differential TFs, providing a basis for the regulation of Hub genes during acute renal transplant rejection and the treatment of rejection.

miRNAs are very powerful gene regulators, and a single miRNA can affect biological functions by interacting with a large number of target genes [37], so miRNAs have attracted the attention of a wide range of scholars. The study on the miRNAs related to Hub gene regulation by the miRDB database showed a total of 90 miRNAs related to CCR2 regulation and a total of 21 miRNAs related to TLR3, among which hsa-miR-8070 can regulate 2 Hub genes simultaneously. The current study has confirmed that miRNA expression might be closely related to the graft function. A multivariate regression analysis of renal transplantation showed that expressions of miR-217 and miR-125b are associated with delayed recovery of graft function [38]. Targeting miRNA expression in the future may have significant potential in protecting graft function.

Drug therapy after renal transplantation is essential because drug intervention can bring better transplant renal function and improve the long-term prognosis of patients. However, there are still some patients with poor efficacy of post-transplantation drug intervention. Therefore, the GeneCards database was utilized to predict the drugs or molecular compounds related to the regulation of Hub genes, with a total of 30 drugs or compounds found to regulate the Hub genes, among which there were 11 related to the regulation of TLR3 and 19 related to the regulation of CCR2, which may be able to bring some help to patients with the long-term use of medication after renal transplantation.

In the study, a comprehensive bioinformatics analysis of peripheral blood transcriptome data for acute renal transplant rejection was performed to construct macrophage-associated biomarkers for acute renal transplant rejection in peripheral blood consisting of CCR2 and TLR3 and explore the associated biological functions and immune cell infiltration levels, which provided a theoretical basis for clinical noninvasive prediction of acute renal transplant rejection. However, there are still some limitations in our study. First, the analysis in our study was only based on transcriptomic data from public databases, without the validation using large samples of clinical cases, which will be the direction of our next research. Second, our analysis was a secondary mining of previously published datasets, which may lead to different conclusions due to different analytical ideas and perspectives. Finally, we validated the Hub gene in this study only by external datasets and rat experiments, and did not test it in human peripheral blood. Also, we did not compare it with commonly used clinical indicators such as serum creatinine, DSA and renal biopsy. Therefore, the diagnostic value of the Hub genes we identified for AR is still in the theoretical stage, and a large number of animal and clinical experiments are still needed for subsequent validation.

## 5. Conclusion

We identified two macrophage-associated Hub genes, CCR2 and TLR3, by comprehensive bioinformatics analysis of peripheral blood data from acute rejection of kidney transplantation.The two Hub genes may have a diagnostic role in kidney transplantation AR, and provide a new target for the diagnosis of kidney transplantation AR. In addition, our findings provide a theoretical basis for the future use of noninvasive assays to predict the occurrence of acute renal transplant rejection.

## Data Availability

The datasets presented in this study can be found in online repositories. The names ofthe repository/repositories and accession number(s) can be found in the article/supplementary material. Transcriptome sequencing results of rat serum are not publicly available due to privacy. The data presented are available on request from the corresponding author.
